# Hepatocyte SHP-1 is a Critical Modulator of Inflammation During Endotoxemia

**DOI:** 10.1038/s41598-017-02512-7

**Published:** 2017-05-22

**Authors:** Anupam Adhikari, Caroline Martel, André Marette, Martin Olivier

**Affiliations:** 1 Department of Medicine, Microbiology and Immunology, Faculty of Medicine, McGill University, Montréal, Québec Canada; 2 The Research Institute of the McGill University Health Centre and Infectious Diseases and Immunity in Global Health Program, Montréal, Québec Canada; 3 Heart and Lung Institute (Laval Hospital), Université Laval, Québec, QC Canada

## Abstract

Liver hepatocytes (Hep) are known to be central players during the inflammatory response to systemic infection. Interestingly, the protein tyrosine phosphatases (PTP) SHP-1, has been recognized as a major regulator of inflammation; however their implication in the control of Hep-mediated inflammatory response is still unknown. To study its implication in the regulation of the Hep-mediated inflammatory response during endotoxemia, Cre-Lox mice with a Hep-specific *Ptpn6* deletion (*Ptpn6* ^*H-KO*^) were injected with LPS. In contrast to the wild-type mice (*Ptpn6* ^*f/f*^) that started to die by 24 hrs post-inoculation, the *Ptpn6* ^*H-KO*^ mice exhibited mortality by 6 hrs. In parallel, higher amounts of metabolic markers, pro-inflammatory mediators and circulating cytokines were detected in *Ptpn6* ^*H-KO*^ mice. Primary Hep obtained from *Ptpn6* ^*H-KO*^, also showed increased secretion of pro-inflammatory cytokines and nitric oxide (NO) comparatively to its wild type (*Ptpn6* ^*f/f*^) counterpart. Pharmacological approaches to block TNF-α and NO production protected both the *Ptpn6* ^*f/f*^ and the *Ptpn6* ^*H-KO*^ mice against deadly LPS-mediated endotoxemia. Collectively, these results establish hepatocyte SHP-1 is a critical player regulating systemic inflammation. Our findings further suggest that SHP-1 activation could represent a new therapeutic avenue to better control inflammatory-related pathologies.

## Introduction

Sepsis is a systemic, hyper-inflammatory immune condition triggered in response to invading pathogens^1^, and responsible for the death of millions of human worldwide^2^. It is characterized by uncontrolled production of pro-inflammatory mediators in the systemic circulation resulting from dysregulated host innate inflammatory response toward infection^3^. Persistence of a high level of pro-inflammatory mediators in the systemic circulation induces multi-organ failure or multi-organ dysfunction syndrome leading to patient death^4^. Sepsis involves excessive production of pro-inflammatory cytokines, such as Tumor necrosis factor alpha (TNF-α)^5^, Interleukin 1 beta (IL-1β)^6^ and Interleukin 6 (IL-6)^7^, which in turn hyper-activate different immune and non-immune cell types for the production of nitric oxide (NO)^8^ and reactive oxygen species (ROS)^9^.

Uncontrolled activation of phagocytes NF-κB and mitogen-activated protein kinase (MAPK) signalling cascades upon bacterial endotoxin stimulation have been found to be critical for the sepsis-induced hyper-production of various inflammatory mediators. For instance, mice deficient for MAPK phosphatase dual specificity phosphatase 1 (DUSP1)^10^ and MAP kinase phosphatase 1 (MKP-1)^11^ showed exacerbated Lipopolysaccharide (LPS)-induced endotoxemia conducting to rapid death and to be paralleled by enhance MAPK signalling pathway activation and augmented pro-inflammatory cytokines secretion^10, 11^. Therefore, modulation of the NF-κB and MAPK pathways may be useful for controlling sepsis-induced pathological manifestations.

Importantly, in addition to phagocytes being involved in the secretion of these inflammatory mediators, it is known that human hepatocytes directly stimulated with bacterial endotoxin can produce large amount of NO and pro-inflammatory cytokines that can significantly contribute to systemic inflammation development^12–14^ establishing liver as a crucial organ for the regulation of sepsis-induced pathology. In this regard, the Signal transducer and activator of transcription 3 (STAT3) signalling axis in hepatocytes has been identified as a profound negative regulator of the sepsis-associated, dysregulated inflammatory response^15^.

In addition to transcriptional regulatory mechanisms, ablation or suppression of selective anti-inflammatory signalling networks in hepatocytes may be important for the development of sepsis. One potential candidate is the protein tyrosine phosphatase SHP-1 since it is highly expressed in hepatocytes^16^ and is known to exert anti-inflammatory effects^17^. SHP-1 is a protein tyrosine phosphatase with two Src homology 2 domains and acts as a critical negative regulator of both innate and acquired immune responses^18, 19^. SHP-1 has been associated with several human inflammatory diseases. Psoriatic inflammatory skin disease patients exhibit a deficiency in the expression of SHP-1 in T cells^20^ and the macrophages of multiple sclerosis patients display SHP-1 deficiency^21^. Moreover, a previous report suggested that altered expression of SHP-1 in mast cells is associated with human allergies and asthmatic disease^22^. SHP-1 signalling within hepatocytes controls a variety of physiological and pathological processes^23, 24^; however, the significance of hepatocyte-specific SHP-1 has not been elucidated under systemic inflammatory conditions.

In the present study, the role of hepatocyte-specific SHP-1 signalling in the regulation of sepsis-induced inflammation was explored. We used hepatocyte-specific SHP-1 deficient mice (*Ptpn6* ^*H-KO*^) and studied the impact of SHP-1 signalling within hepatocytes in murine model of systemic inflammation induced by LPS. Compared to their wild-type counterparts (*Ptpn6* ^*f/f*^), the LPS-injected *Ptpn6* ^*H-KO*^ mice exhibited increased mortality in association with higher amounts of lipopolysaccharide binding protein (LBP), serum amyloid A (SAA), and NO in the serum, and prolonged activation of MAPK in the liver, leading to a greater production of various pro-inflammatory cytokines. This study reveals a novel role of hepatocyte SHP-1 in controlling the production of inflammatory mediators during systemic inflammation, thus suggesting that the PTPase is a key player of the close link between hepatocyte metabolism and the immune system.

## Results

### *Ptpn6*^*H-KO*^ mice are highly susceptible to LPS-induced endotoxemia and multiple organ failure

**Figure 1:**
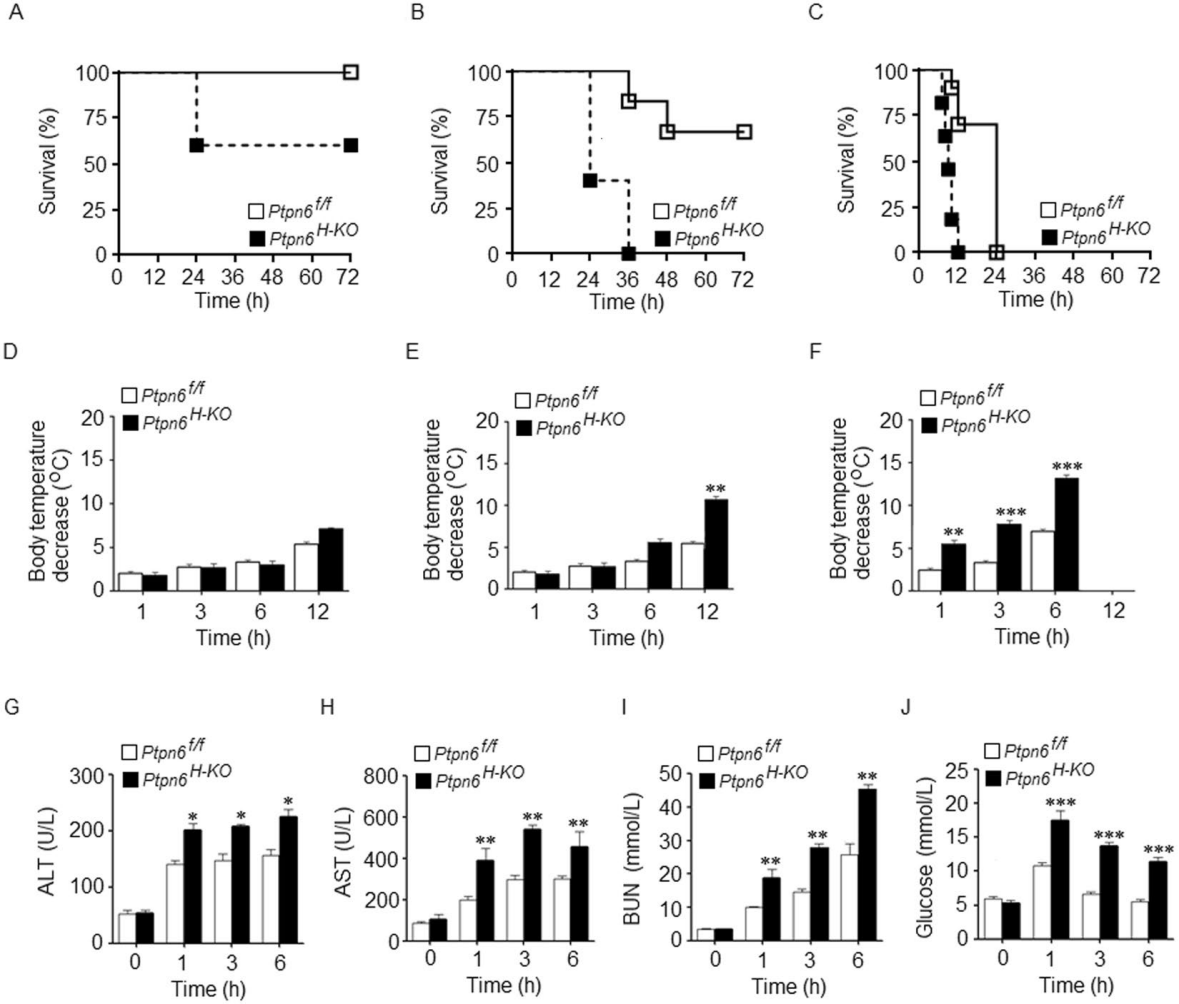
*Ptpn6* ^*H-KO*^ mice are highly susceptible to multiple organ failure and endotoxemia. Survival curve of *Ptpn6* ^*f/f*^ (WT) and *Ptpn6* ^*H-KO*^ (KO) mice after LPS challenge of (**A**) 5 mg/kg body weight (*n* = 11 for WT mice and *n* = 10 for KO mice), (**B**) 10 mg/kg body weight (*n* = 11 for WT mice and *n* = 12 KO mice), and (**C**) 30 mg/kg body weight (*n* = 17 for WT mice and *n* = 18 for KO mice). *P* ≤ 0.03 for 10 mg/kg body weight and *P* ≤ 0.005 for 30 mg/kg body weight. Decrease in body temperature of *Ptpn6* ^*f/f*^ and *Ptpn6* ^*H-KO*^ mice after LPS challenge of (**D**) 5 mg/kg body weight, (**E**) 10 mg/kg body weight, and (**F**) 30 mg/kg body weight. Significant differences in body temperature reduction between *Ptpn6* ^*H-KO*^ and *Ptpn6* ^*f/f*^ mice at the indicated time points denoted with **P ≤ 0.05 and ***P ≤ 0.01. Serum levels of (**G**) ALT, (**H**) AST, (**I**) BUN and (**J**) Glucose in *Ptpn6* ^*f/f*^ and *Ptpn6* ^*H-KO*^ mice after LPS challenge (30 mg/kg body weight). Significant differences between LPS challenge *Ptpn6* ^*H-KO*^ and *Ptpn6* ^*f/f*^ mice at the indicated time points denoted with *P ≤ 0.05, **P ≤ 0.01 and ***P ≤ 0.01. Data are presented as mean ± SEM (*n* = 3–6 in each group).

The liver is the central regulator of inflammation^25^ and the protein tyrosine phosphatase SHP-1 plays a crucial role in regulating inflammation^18, 19^. However, the function of hepatocyte-specific SHP-1 during LPS-induced endotoxic shock has not been studied to date. Therefore, we examined whether hepatocyte SHP-1 had any influence on the regulation of systemic inflammation during endotoxemia. We induced endotoxemia in the *Ptpn6* ^*f/f*^ and *Ptpn6* ^*H-KO*^ mice by injecting various doses of LPS intraperitoneally and measured survival and body temperature as indicators of LPS-mediated intoxication. At a relatively low dose of LPS challenge (5 mg/kg body weight), there was no statistically significant difference in survival (Fig. 1A) and body temperature (Fig. 1D) between the *Ptpn6* ^*H-KO*^ and *Ptpn6* ^*f/f*^ mice. Although, the survival of the *Ptpn6* ^*H-KO*^ mice was affected to a greater extent compared to the *Ptpn6* ^*f/f*^ mice. In contrast, we observed that at higher dose of LPS challenge (10 mg/kg body weight), all of the *Ptpn6* ^*H-KO*^ mice died within 36 h. At the same dose of LPS challenge, nearly 70% of the *Ptpn6* ^f/f^ mice survived past 72 h (Fig. 1B). Moreover, we observed a significant reduction in body temperature in the *Ptpn6* ^*H-KO*^ mice compared to the *Ptpn6* ^*f/f*^ mice at the same dose (10 mg/kg body weight) of LPS challenge (Fig. 1E). When the dose of LPS was further increased (30 mg/kg body weight), all the *Ptpn6* ^*H-KO*^ mice died within 12 hours, whereas most of the *Ptpn6* ^*f/f*^ mice survived f or greater than 12 hours (Fig. 1C). Furthermore, at this higher dose of LPS, we observed a significant decrease in the body temperature of the *Ptpn6* ^*H-KO*^ mice compared to the *Ptpn6* ^*f/f*^ mice starting as early as 1 h post-LPS challenge (Fig. 1F).

To examine the effects of SHP-1 deficiency in hepatocytes on the function of key organs, we measured the amount of alanine aminotransferase (ALT) and aspartate transaminase (AST) in the serum to determine liver function and blood urea nitrogen (BUN) to determine renal function in the *Ptpn6* ^*H-KO*^ and *Ptpn6* ^*f/f*^ mice following LPS challenge (30 mg/kg body weight). Interestingly, we observed that LPS induced significantly higher amounts of ALT, AST (Fig. 1G,H) and BUN (Fig. 1I) in the *Ptpn6* ^*H-KO*^ mice compared to that of the *Ptpn6* ^*f/f*^ mice. In addition, LPS challenge induced hyperglycemia in both the *Ptpn6* ^*H-KO*^ and *Ptpn6* ^*f/f*^ mice during early time points (1 hour); however, it was only sustained at the later time points in the *Ptpn6* ^*H-KO*^ mice (Fig. 1J).

Collectively, our results indicate that the *Ptpn6* ^*H-KO*^ mice are highly susceptible to multiple organs failure compared to the *Ptpn6* ^*f/f*^ mice after LPS administration and that hepatocyte SHP-1 plays a crucial role in regulating the sensitivity of mice to endotoxemia.

### LPS challenge induced an exacerbated inflammatory response in *Ptpn6*^*H-KO*^ mice by modulating MAPK activation

**Figure 2:**
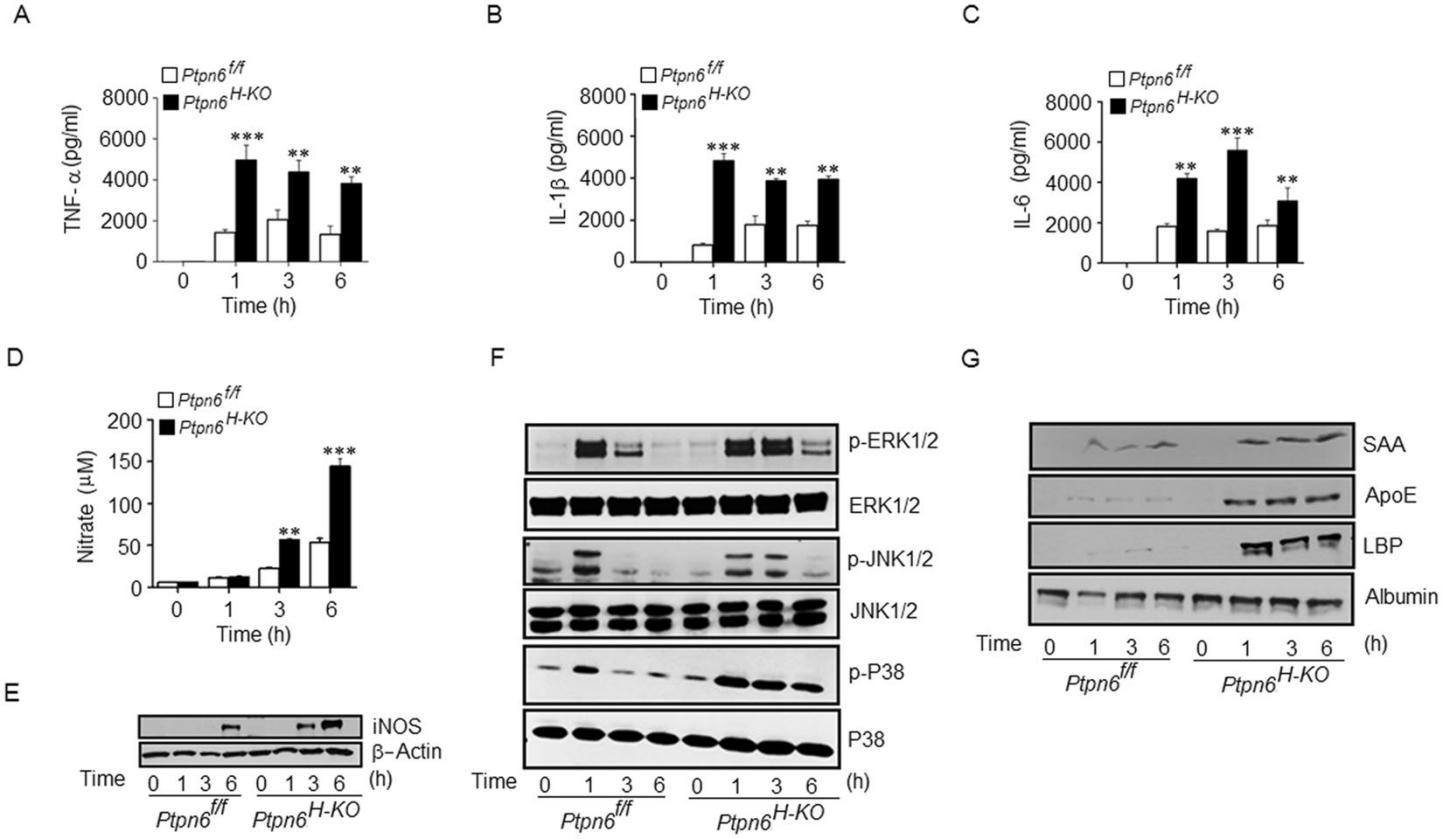
LPS challenge induced an exacerbated inflammatory response in the *Ptpn6* ^*H-KO*^ mice by modulating MAPK activation. Serum concentration of (**A**) TNF-α (**B**) IL-1β, (**C**) IL-6 and (**D**) nitric oxide in *Ptpn6* ^*H-KO*^ and *Ptpn6* ^*f/f*^ mice after LPS challenge (30 mg/kg body weight). Data are presented as mean ± SEM (*n* = 6 in each group) **P ≤ 0.05 and ***P ≤ 0.01. Liver levels of (**E**) iNOS and (**F**) phospho-JNK1/2, phospho-ERK1/2 and phospho-p38, and serum levels of (**G**) SAA, ApoE and LBP in *Ptpn6* ^*H-KO*^ and *Ptpn6* ^*f/f*^ mice after LPS challenge (30 mg/kg body weight). The blots shown here are cropped from full length gel. Full-length blots are included in the Supplementary Information file. (**E**) iNOS in Supplementary Information 1 (**F**) phospho-JNK1/2, phospho-ERK1/2 and phospho-p38 in Supplementary Information 2 and serum levels of (**G**) SAA, ApoE and LBP in *Ptpn6* ^*H-KO*^ and *Ptpn6* ^*f/f*^ mice Supplementary Information 3.

It is now well established that the lethality of endotoxemia is due to the dysregulated over-production of different pro-inflammatory mediators, including different cytokines and nitric oxide (NO). Therefore, we analyzed the expression of different pro-inflammatory mediators in the serum of the *Ptpn6* ^*H-KO*^ and *Ptpn6* ^*f/f*^ mice at different time points following LPS challenge (30 mg/kg body weight). We observed that the *Ptpn6* ^*H-KO*^ mice produced higher levels of TNF-α, IL-1β and IL-6 as compared to their *Ptpn6* ^*f/f*^ counterparts at all-time points in response to LPS challenge (Fig. 2A–C). A very significant increase in the plasma nitrate levels was measured in the *Ptpn6* ^*H-KO*^ mice compared to that of the *Ptpn6* ^*f/f*^ mice at 3 and 6 h following LPS challenge (Fig. 2D). In addition, we observed a significant augmentation of inducible nitric oxide synthase (iNOS) expression in the liver of the *Ptpn6* ^*H-KO*^ mice compared to that of the *Ptpn6* ^*f/f*^ mice during LPS challenge (Fig. 2E). Since the activation of MAPK is a crucial determinant for the production of LPS-induced inflammatory mediators^9^, we examined whether hepatocyte-specific SHP-1 deficiency had any influence on the regulation of MAPK signalling pathways during endotoxin shock. Interestingly, we observed that LPS induced rapid phosphorylation or activation of various MAPKs (p38, JNK1/2 and ERK1/2) in the liver of both the *Ptpn6* ^*H-KO*^ and *Ptpn6* ^*f/f*^ mice immediately after challenge (1 hour). The phosphorylation of these MAPKs diminished with time (3 and 6 hours) in the *Ptpn6* ^*f/f*^ mice, but the *Ptpn6* ^*H-KO*^ mice exhibited a sustained phosphorylation of these MAPKs even 3 h after LPS challenge (Fig. 2F). Previous reports suggested that acute phase proteins, including LBP^26^, SAA^27^ and Apolipoprotein E (ApoE)^28^, produced by the liver during inflammation play a stimulatory role in enhancing the extent and pathology of inflammation. Therefore, we determined whether SHP-1 deficiency in hepatocytes modulated the expression of LBP, SAA and ApoE after LPS challenge. We observed a significant enhancement in the amount of LBP, SAA and ApoE protein at 1, 3 and 6 hours post-LPS challenge in the *Ptpn6* ^*H-KO*^ mice compared to the *Ptpn6* ^*f/f*^ mice, as evidenced by whole serum western blot analysis (Fig. 2G).

Therefore, these observations suggest that hepatocyte-specific SHP-1 has an important role in regulating the production of inflammatory mediators by modulating MAPK activation in the liver during endotoxemia and in regulating acute phase protein secretion by the liver during LPS-induced inflammation.

### LPS challenge induced greater neutrophil recruitment and NO production in the peritoneum of *Ptpn6*^*H-KO*^ mice

**Figure 3:**
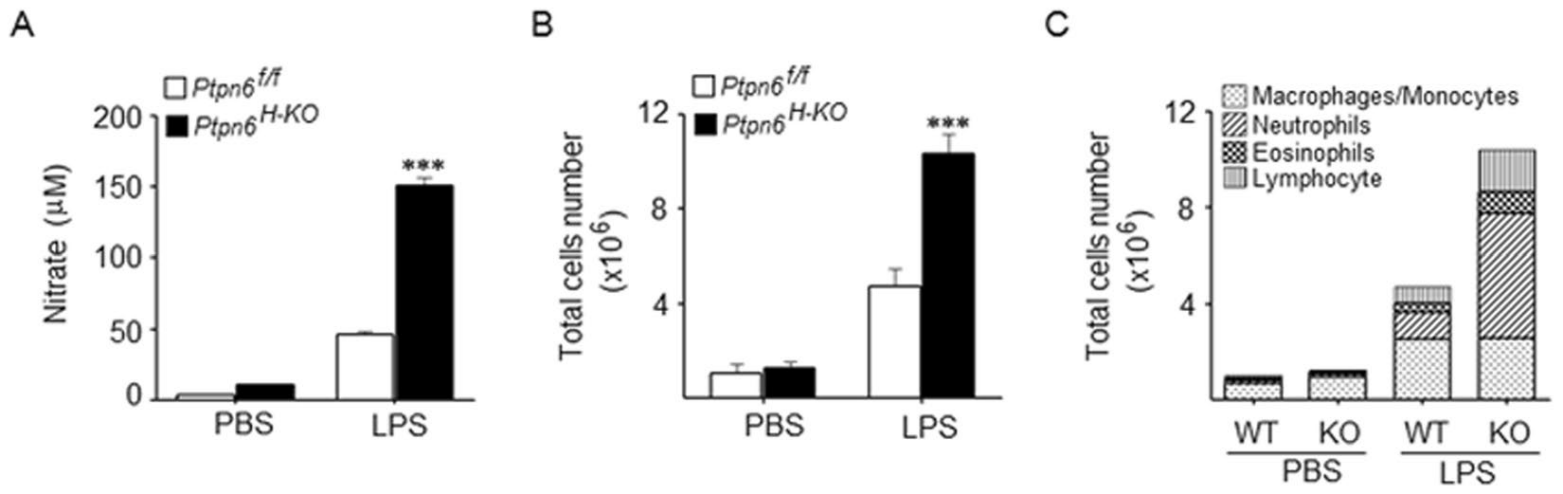
Nitric oxide, total cell number and neutrophil recruitment in peritoneum of *Ptpn6* ^*H-KO*^ mice were significantly higher during LPS challenge. (**A**) NO production, (**B**) total cell count, and (**C**) neutrophil accumulation in the peritoneal fluid of *Ptpn6* ^*H-KO*^ and *Ptpn6* ^*f/f*^ mice after LPS challenge (10 mg/kg body weight). Data are presented as mean ± SEM (*n* = 6 in each group) ***P ≤ 0.01.

LPS intoxication enhances peritoneal cavity inflammation through the recruitment of various inflammatory cells and their subsequent activation to produce high levels of inflammatory mediators^29^. In an attempt to characterize this response further, we studied NO generation, and total cell and neutrophil recruitment to the peritoneal cavity of the *Ptpn6* ^*H-KO*^and *Ptpn6* ^*f/f*^ mice 6 h after LPS challenge. We detected a significantly higher amount of NO in the peritoneal lavage of the *Ptpn6* ^*H-KO*^ mice compared to that of the *Ptpn6* ^*f/f*^ mice 6 h post-LPS challenge (Fig. 3A). In addition, the total number of cells recruited to the peritoneal cavity of the *Ptpn6* ^*H-KO*^ mice was found to be significantly higher compared to the *Ptpn6* ^*f/f*^ mice after 6 h of LPS challenge (Fig. 3B). Moreover, the amount of neutrophils in the peritoneal fluid of the *Ptpn6* ^*H-KO*^ was found to be markedly higher compared to the *Ptpn6* ^*f/f*^ mice following 6 h of LPS challenge (Fig. 3C). Collectively, our observations indicate that hepatocyte specific SHP-1 has a crucial role in neutrophil immigration to the peritoneum during endotoxemia.

### *Ptpn6*^*H-KO*^ hepatocytes produce higher levels of inflammatory mediators during LPS challenge *in vitro*

**Figure 4:**
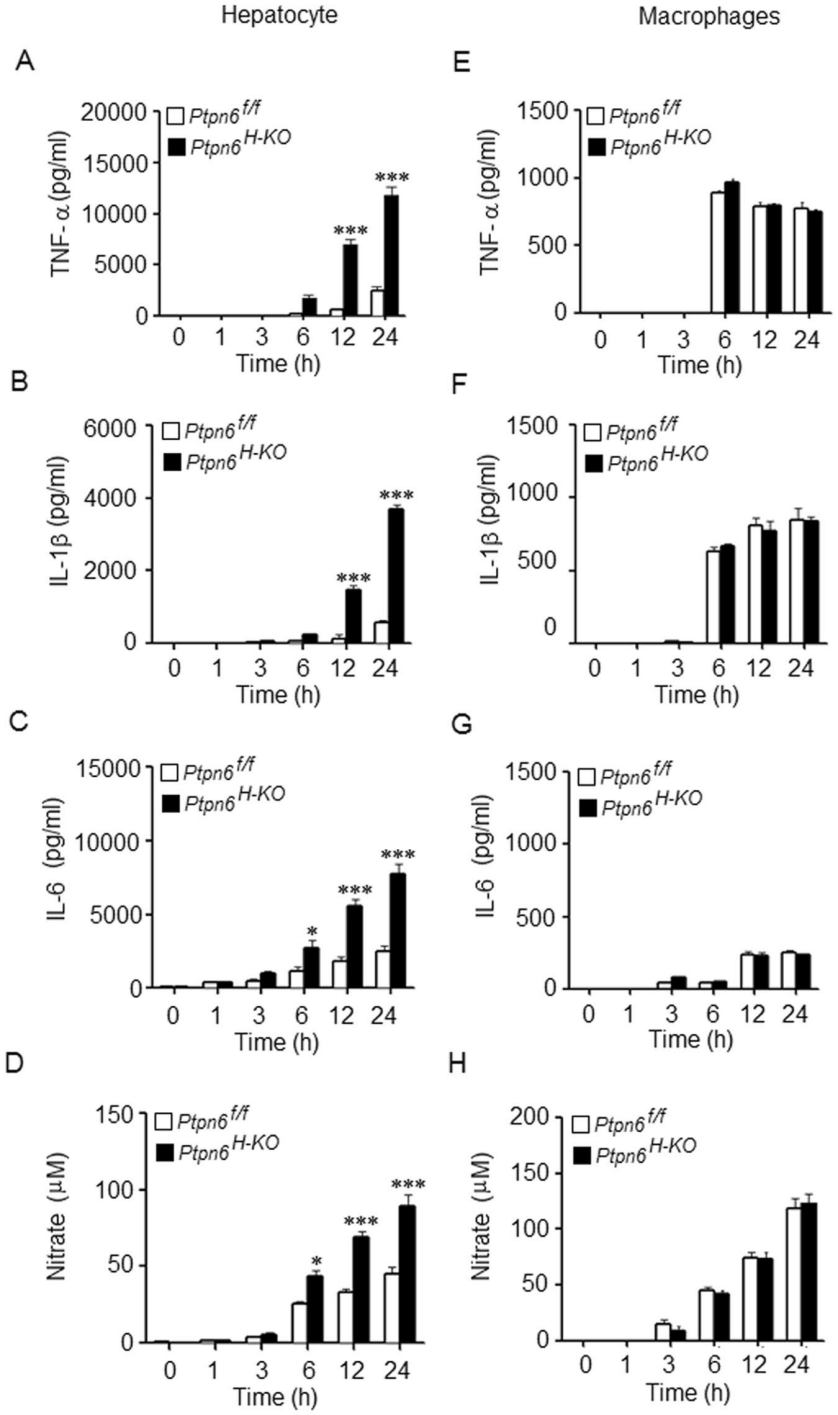
LPS induced inflammatory mediators in hepatocytes of *Ptpn6* ^*H-KO*^ mice. Production of (**A**) TNF-α, (**B**) IL-1β, (**C**) IL-6 and (**D**) Nitric oxide by hepatocytes isolated from *Ptpn6* ^*H-KO*^ and *Ptpn6* ^*f/f*^ mice after LPS stimulation (1 μg/ml). Data are presented as mean ± SEM of three samples in three independent experiments. Production of (**E**) TNF-α, (**F**) IL-1β, (**G**) IL-6 and (**H**) nitric oxide by peritoneal macrophages isolated from *Ptpn6* ^*H-KO*^ and *Ptpn6* ^*f/f*^ mice after LPS stimulation (1 μg/ml). Data are presented as mean ± SEM of three samples in three independent experiments. *P ≤ 0.05, **P ≤ 0.01 and ***P ≤ 0.001.

To strengthen our *in vivo* findings, we carried out further experiments to characterize the consequences of SHP-1 deficiency on the hepatocyte-mediated production of different inflammatory mediators *in vitro*. We observed that LPS (1 µg/ml) induced a higher level of TNF-α, IL-1β and IL-6 expression in hepatocytes from *Ptpn6* ^*H-KO*^ mice compared to hepatic cells from *Ptpn6* ^*f/f*^ mice at 12 h and 24 h (Fig. 4A–C). Furthermore, hepatocytes from the *Ptpn6* ^*H-KO*^ mice exhibited a very significant increase in NO production compared to hepatocytes of the *Ptpn6* ^f/f^ mice, especially at 12 and 24 h post-LPS stimulation (Fig. 4D). However, we observed no significant difference in pro-inflammatory cytokine production and NO generation in the peritoneal macrophages isolated from both *Ptpn6* ^*H-KO*^ and *Ptpn6* ^*f/f*^ mice following LPS stimulation (Fig. 4E–H). Collectively, our observations indicate that hepatocyte SHP-1 has a pivotal role in modulating the inflammatory response to LPS.

### LPS induced sustained MAPK activation in the hepatocytes of *Ptpn6*^H-KO^ mice *in vitro*

**Figure 5:**
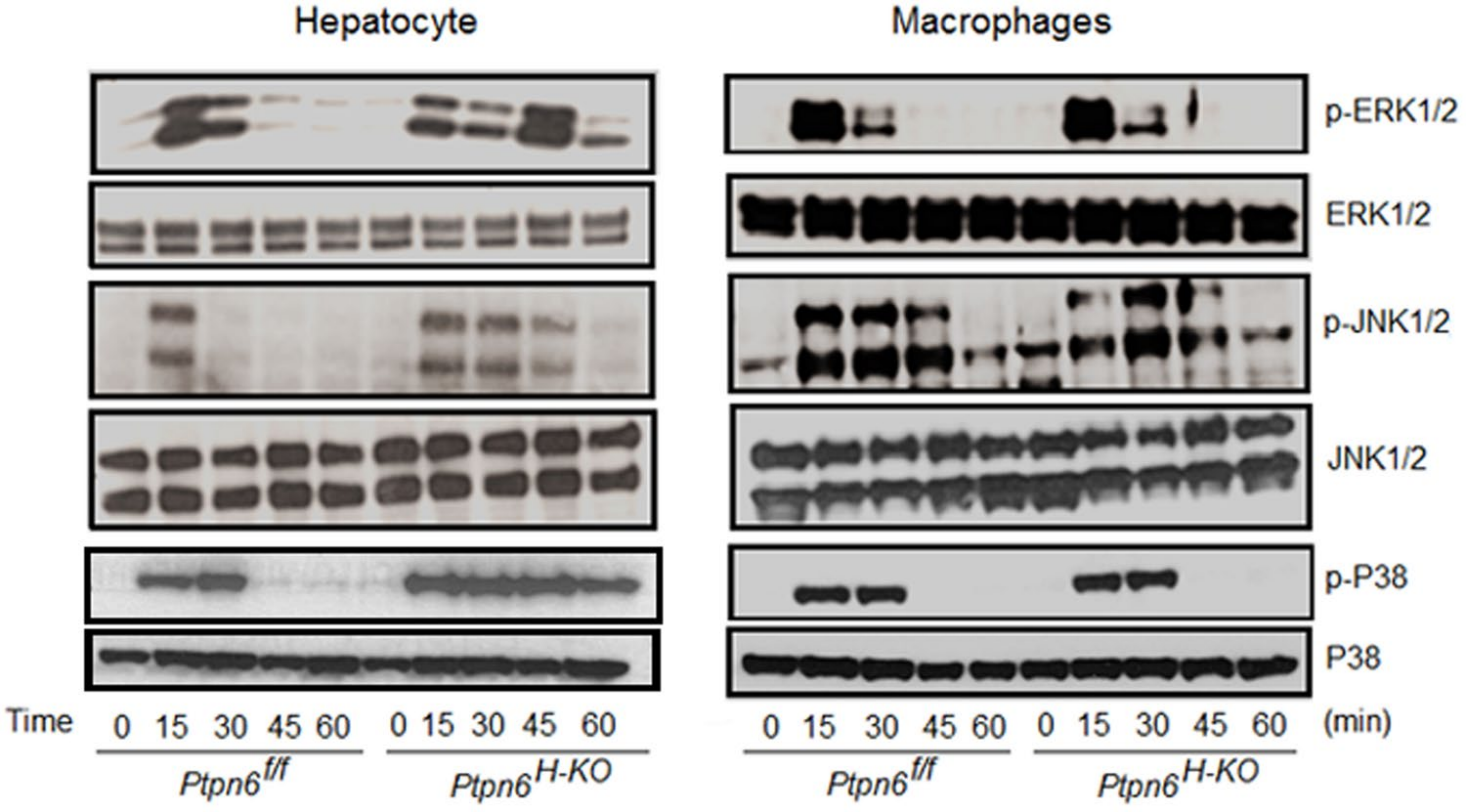
Attenuation of MAPKs activation in *Ptpn6* ^*f/f*^ hepatocyte after LPS stimulation. Western blot of phospho-ERK1/2, phospho-JNK1/2 and phospho-p38 in hepatocytes and peritoneal macrophages isolated from *Ptpn6* ^*H-KO*^ and *Ptpn6* ^*f/f*^ mice treated with LPS (1 μg/ml). The blots shown here are cropped from full length gel. Full-length blots are included in the Supplementary Information file. Phospho-ERK1/2, phospho-JNK1/2 and phospho-p38 in hepatocytes in Supplementary Information 4, Phospho-ERK1/2, phospho-JNK1/2 and phospho-p38 in peritoneal macrophages in Supplementary Information 5.

To validate our *in vivo* findings, we performed experiments to examine whether SHP-1 deficiency had any significant influence on the activation of MAPK in hepatocytes after LPS stimulation *in vitro*. Rapid phosphorylation of MAPK induced by LPS was significantly abrogated in hepatocytes of *Ptpn6* ^*f/f*^ mice compared to that of hepatocytes of *Ptpn6* ^*H-KO*^ mice at later time points (45 and 60 min) (Fig. 5). To further confirm that the negative regulation of MAPK activation during LPS stimulation was solely dependent on the deficiency of SHP-1, we analyzed the activation of different MAPK in the peritoneal macrophages of the *Ptpn6* ^*H-KO*^ and *Ptpn6* ^*f/f*^ mice following LPS stimulation. We observed no significant difference in the status of MAPK activation in the peritoneal macrophages isolated from the *Ptpn6* ^*H-KO*^ and *Ptpn6* ^*f/f*^ mice at early as well as late time points of LPS stimulation (Fig. 5). Thus, these results clearly demonstrate that hepatocyte SHP-1 is a crucial negative regulator of the activation of various MAPK in response to LPS stimulation.

### Treatment with TNF-α inhibitor pentoxifylline and/or iNOS/NO inhibitor aminoguanidine rescued *Ptpn6*^*H-KO*^ mice against deadly LPS challenge

**Figure 6:**
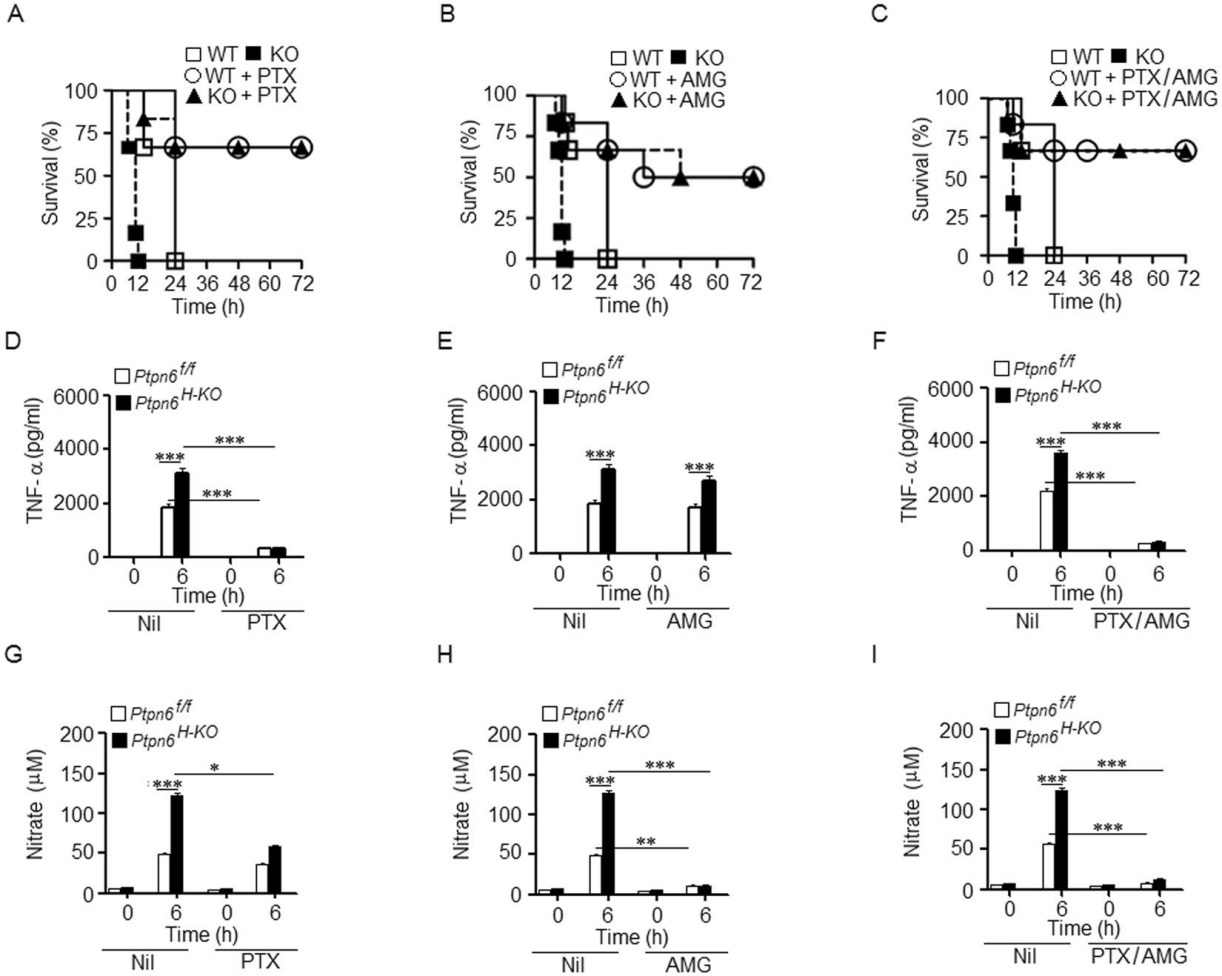
TNF-α and/or iNOS/NO inhibitors rescued *Ptpn6* ^*H-KO*^ challenged with LPS. Survival curve of *Ptpn6* ^*H-KO*^ and *Ptpn6* ^*f/f*^ mice treated with (**A**) pentoxifylline, (**B**) aminoguanidine or (**C**) their combination after LPS challenge (30 mg/kg body weight). Kaplan-Meier analysis demonstrates a significant difference in the survival of both *Ptpn6* ^*H-KO*^ and *Ptpn6* ^*f/f*^ mice treated with pentoxifylline, aminoguanidine or their combination compared to no treatment. Serum levels of TNF-α in *Ptpn6* ^*H-KO*^ and *Ptpn6* ^*f/f*^ mice treated with (**D**) pentoxifylline, (**E**) aminoguanidine or (**F**) their combination after LPS challenge (30 mg/kg body weight). Serum levels of NO in *Ptpn6* ^*H-KO*^ and *Ptpn6* ^*f/f*^ mice treated with (**G**) pentoxifylline, (**H**) aminoguanidine or (**I**) their combination after LPS challenge (30 mg/kg body weight). Data are presented as mean ± SEM, *n* = 6. *P ≤ 0.05, **P ≤ 0.01 and ***P ≤ 0.001.

Our previous data indicated that LPS challenge induces a larger amount of TNF-α and NO in *Ptpn6* ^*H-KO*^ mice as compared to *Ptpn6* ^*f/f*^ mice (Fig. 2A,D). Therefore, we pre-treated the *Ptpn6* ^*H-KO*^ and *Ptpn6* ^*f/f*^ mice with pentoxifylline (PTX) (TNF-α inhibitor), aminoguanidine (AMG) (iNOS inhibitor), or both to determine whether the elevated levels of TNF-α and/or NO were responsible for the increased death of the *Ptpn6* ^*H-KO*^ mice following LPS challenge. Interestingly, we observed that pentoxifylline treatment rescued approximately 70% of the *Ptpn6* ^*H-KO*^ mice (Fig. 6A), that aminoguanidine treatment rescued approximately 50% of the *Ptpn6* ^*H-KO*^ mice (Fig. 6B) while the combined treatment was found to rescue approximately 70% of the *Ptpn6* ^*H-KO*^ mice (Fig. 6C). In addition, pentoxifylline treatment significantly decreased the concentration of TNF-α (Fig. 6D) and other cytokines (supplementary Fig. 1A,B) in the serum. Moreover, the concentration of NO in serum was moderately changed by pentoxifylline treatment (Fig. 6G). In contrast, aminoguanidine treatment significantly decreased the concentration of NO in the serum (Fig. 6H), but the concentration of TNF-α (Fig. 6E) and other cytokines (supplementary Fig. 2A,B) in the serum remained unaltered. The combination of pentoxifylline and aminoguanidine significantly decreased the concentration of TNF-α (Fig. 6F), NO (Fig. 6I) and other pro-inflammatory cytokines in the serum (supplementary Fig. 3A,B). Furthermore, we observed no significant difference in the body temperature between the *Ptpn6* ^*H-KO*^ and the *Ptpn6* ^*f/f*^ mice that were treated with aminoguanidine, pentoxifylline or their combination (supplementary Fig. 4A–C)

Therefore, these findings demonstrate that the inhibition of TNF-α or NO release by their inhibitors can abrogate the increased sensitivity of the *Ptpn6* ^*H-KO*^ mice to LPS-induced endotoxemia.

## Discussion

The pathophysiology of sepsis involves dysregulation of the inflammatory response, leading to an imbalance between pro- and anti-inflammatory mediators^30^. The liver is an immunocompetent organ that plays a key role in regulating sepsis-induced pathology^31^. SHP-1 deficiency is associated with several types of chronic inflammatory disorders and malignancies^20–22^, which has raised substantial interest in this phosphatase. SHP-1 signalling within hepatocytes controls a variety of physiological and pathological processes^23, 24^; however, the significance of hepatocyte-specific SHP-1 has not yet been elucidated under LPS-induced, systemic inflammatory conditions.

Herein we report the first demonstration that hepatocyte SHP-1 deficiency leads to exacerbated levels of circulating inflammation-related biomarkers, pro-inflammatory cytokines and mortality upon LPS challenge, revealing the cardinal role played by hepatic SHP-1 in the regulation of inflammatory response during systemic inflammation. More precisely, we found that SHP-1 in hepatocytes plays a critical role in the negative regulation of MAPK signalling -known to be involved in pro-inflammatory cytokines production by these cells- as revealed by the rapid and prolonged activation of MAPK upon LPS bacterial endotoxin challenge *in vivo*. In parallel, the increased circulating LPS-induced pro-inflammatory cytokines measured in *Ptpn6* ^*H-KO*^ mice versus *Ptpn6* ^*f/f*^ mice further reinforced the cardinal role of SHP-1 as regulator of hepatocyte in septic inflammation.

Importantly, it is known that during LPS- induced endotoxemia, animal death is accompanied by major dysfunctions of vital organs that can be monitored by measuring levels of circulating ALT, AST, BUN and glucose^32^ of utmost interest, the extreme sensitivity of *Ptpn6* ^*H-KO*^ mice to LPS-mediated endotoxemia and death was also reflected by rapid and intense collapse of organs’ function as exacerbated levels of these pathological markers were detected. This strongly suggests that hepatocyte SHP-1 acts as a vital suppressor of inflammatory responses, thereby protecting the host from shock, multiple organ failure, and mortality after exposure to LPS.

However, the mechanism whereby hepatocyte controls LPS-induced hyper-inflammation and multiple organ failure in the host is still only partially elucidated. Our findings have established that the MAPK signalling pathways regulating pro-inflammatory cytokines and NO synthesis in hepatocytes during LPS challenge is not properly controlled in the absence of SHP-1, therefore resulting in the overproduction of pro-inflammatory mediators. A previous report suggested that MAPK, mainly JNK1/2 and p38, control TNF-α biosynthesis by stabilizing TNF-α mRNA and by augmenting its translation^33^. Thus, the sustained activation of JNK1/2 and p38 in the liver of the *Ptpn6* ^*H-KO*^ mice after LPS challenge could explain the prolonged TNF-α biosynthesis in the *Ptpn6* ^*H-KO*^ mice. A likely explanation for the shock and multiple organ failure might be that the increased amount of TNF-α triggers a considerable elevation in NO synthase activity, leading to multiple organ failure. Of interest, previous studies using MAPK phosphatase (DUSP1 and MKP-1) KO mice^10, 11^ reported that their sensitivity and death were also augmented, further supporting our observations. But of utmost importance, and a key difference from those previous studies that used systemic KO mice, our hepatocyte specific SHP-1 KO mice revealed the extent to which liver hepatocytes are critical in the regulation of septic inflammation. Additionally, by focusing our study on hepatocytes, we also revealed that SHP-1 is an important regulator of various acute phase proteins known to be important biomarkers during inflammation^26–28^.

Liver is the first organ being encountered by the blood draining the peritoneum and endotoxin induced peritonitis -known to be a common cause of sepsis^34^. In this regard, we showed that in absence of hepatocyte SHP-1, LPS-mediated inflammatory cells recruitment in the peritoneal cavity was also exacerbated, in particular for neutrophils. These data further revealed that abnormal recruitment of inflammatory cells in peritoneal environment during endotoxemia could be an important step leading to augmented amount of pro-inflammatory cytokines detected in animals. Whereas previous studies have suggested that the response of hepatocytes to LPS is complex, requiring cell-cell interaction between hepatocytes and other cells present in the liver, such as Kupffer cells and sinusoidal endothelial cells^35^. However, our data indicate, suggested by others^36^, that LPS can directly stimulate hepatocytes to release various inflammatory related mediators. In fact, using an *in vitro* approach, we observed that primary hepatocytes isolated from *Ptpn6* ^*H-KO*^ and *Ptpn6* ^*f/f*^ mice and subjected to LPS stimulation in culture showed marked modulation of MAPKs and secretion of major pro-inflammatory cytokines. These findings establish a cell-autonomous role for hepatocytes and that other cells are not essential to endotoxin stimulation in the context of our studies. Importantly, here again, the greater sensitivity of *Ptpn6* ^*H-KO*^ mice hepatocytes toward LPS was reflected by rapid and sustained phosphorylation of the three major MAPK subfamilies (ERK1/2, JNK1/2, and p38) and the greater production of pro-inflammatory cytokines comparatively to the *Ptpn6* ^*f/f*^ mice. Notably, the finding of increased cytokine expression in relation to SHP-1 deficiency *in vitro* was in accordance with our *in vivo* studies where serum levels of TNF-α, IL-1β, and IL-6 were increased in the *Ptpn6* ^*H-KO*^ mice compared to the *Ptpn6* ^*f/f*^ mice during endotoxemia. Furthermore, the *in vitro* demonstration that there was no significant difference in MAPK activation or in the generation of inflammatory mediators by peritoneal macrophages isolated from both *Ptpn6* ^*H-KO*^ and *Ptpn6* ^*f/f*^ mice in response to LPS clearly demonstrate that hepatocyte SHP-1 is a key negative regulator of liver MAPK activation and consequent functional activation such as attenuation of various inflammatory mediators in response to bacterial endotoxin.

TNF-α is known to be a major proinflammatory mediator of endotoxemia^37^ enhancing the pathophysiological response of sepsis by inducing the release of active substances, such as other proinflammatory cytokines and NO^38^. As mentioned earlier, we have observed a strong correlation between exacerbated TNF-α/NO production and rapid death of *Ptpn6* ^*H-KO*^ mice over more resistant *Ptpn6* ^*f/f*^ mice. Using pentoxifylline -a TNF-α inhibitor- and aminoguanidine -a NO inhibitor- alone or in combination, we tested the importance of these latters and their correlation with the rapid death of our *Ptpn6* ^*H-KO*^ mice. We found that pentoxifylline afforded equal significant protection against LPS-induced shock in both *Ptpn6* ^*H-KO*^ and *Ptpn6* ^*f/f*^ mice, and that the TNFα antagonizing effect of pentoxifylline was also associated with the reduction of other injurious circulating pro-inflammatory cytokines, namely IL-6 and IL-1β. Additionally, aminoguanidine treatment was found to protect against endotoxemia by attenuating NO production in both *Ptpn6* ^*H-KO*^ and *Ptpn6* ^*f/f*^ mice. The combination of both inhibiting molecules, while exerting protection against LPS-induced endotoxemia, did not show any synergism, indicating that the pathophysiology of sepsis in the *Ptpn6* ^*H-KO*^ mice was not strictly dependent on TNF-α or NO.

In conclusion, this study provided strong *in vivo* and cellular evidences that hepatocyte SHP-1 plays a cardinal role in the production of inflammatory mediators that contribute to endotoxemia. Our data further suggest that the development of anti- endotoxemia therapy concurring to induce SHP-1 activity could represent a new avenue to consider, as actual therapies mainly focusing at specifically blocking TNF-α and other pro-inflammatory cytokines showed limited successes.

## Materials and Methods

### Animals and Genotyping

Mice C57BL/6J background (6–8 weeks old) was housed under controlled temperature (23 °C) and a 12-hour light/dark cycle with water and food in pathogen-free condition. All research involving mice was carried out according to the regulations of the Canadian Council of Animal Care and was approved by the McGill University Animal Care Committee under ethics protocol number 5925. Mice were euthanized at established humane endpoints using CO_2_ asphyxiation followed by cervical dislocation or by using isoflurane if perfusion was performed.

Hepatocyte-specific SHP-1 knockout mice (*Ptpn6* ^*H-KO*^) were generated on a pure C57/BL6 background by crossing mice homozygous for floxed SHP-1 (*Ptpn6* ^*f/f*^)^39^ with Alb-Cre mice (B6.Cg-Tg[Alb-cre]21Mgn/J, stock 3574; (The Jackson Laboratory). Genomic DNA was extracted from ear samples using the DNA RED Extract-N-Amp PCR kit (Sigma), and genotyping was performed as described elsewhere^23, 24^.

### LPS injection and endotoxemia

*Ptpn6* ^*f/f*^ and *Ptpn6* ^*H-KO*^ mice were injected intraperitoneally (i.p.) with pyrogen-free PBS or the LPS derived from Escherichia coli Serotype 055:B5 (Sigma, St. Louis) dissolved in pyrogen-free PBS (5, 10 and 30 mg/kg body weight). In a separate experiment, an inhibitor of TNF-α, Pentoxifylline was given (i.p.) at a dose of 150 mg/kg 1 hour before LPS challenge^40^. In another experiment, mice were treated twice a day with the iNOS inhibitor amino guanidine hemisulfate salt (Sigma) 8 mg dissolved in 100 µl PBS as described^41^. Survival of the mice was monitored every 3 h for 72 h. Body temperature of the mice was measured at indicated time points using a thermometer. For serum cytokine and nitric oxide assay, sera were collected at 0, 1, 3 and 6 h after the LPS injection.

### Blood Biochemistry in LPS treated mice

After the intraperitoneal injection of LPS (30 mg/kg body weight), blood samples were obtained from the tail vein at 0, 1, 3 and 6 hours post-inoculation to measure different parameter (blood glucose, blood alanine aminotransferase (ALT), blood aspartate transaminase (AST) and blood urea nitrogen (BUN). Levels of serum glucose, ALT, AST and BUN were measured with a VITROS 250/350/950/5,1 FS and 4600 Chemistry Systems and the VITROS 5600 Integrated System made by Ortho Clinical Diagnostics.

### Isolation and Culture of Mouse Primary Hepatocytes

Livers were perfused in anesthetized mice and primary Hepatocytes were isolated from *Ptpn6* ^*f/f*^ and *Ptpn6* ^*H-KO*^ mice as previously described^42^.

### Preparation of Mouse Peritoneal Macrophage

To isolate peritoneal macrophages, peritoneal exudates cells were isolated from the peritoneal cavity with cold PBS and used the adherent cells as peritoneal macrophages^43^ for further experiments.

### Measurement of Nitric oxide and Cytokines

Hepatocytes and Peritoneal macrophages isolated from *Ptpn6* ^*H-KO*^ and *Ptpn6* ^*f/f*^ mice were stimulated with LPS (1 µg/ml) for different time points (1, 3, 6, 12, and 24 h). Pro-inflammatory cytokines in the culture supernatants and serum were measured by ELISA. TNF-α, IL-1β and IL-6 were measured by ELISA according to the manufacturer’s instructions (SET TO GO kit, eBiosciences, San Diego, CA, USA). Concentration of NO was measured using Griess reagents as previously described^44^.

### Peritoneal lavage

Mice receiving LPS (10 mg/kg body weight) or PBS by i.p. injection were euthanized after 6 h, and total cell counts were made for lavage fluid using a haemocytometer. Cell suspension applied onto a microscope glass-slide using a Cytospin apparatus. The slide was then stained using the Diff-Quik stain (Siemens Healthcare, Newark, DE, USA) and blind differential counting was performed on these slides by microscopy.

### Western blotting

Western blotting was performed as previously described^44^. List of antibodies used: Actin (Sigma, A5316); Anti-phospho-JNK1/2 (Cell signalling, Ipswich, MA, USA), Anti-phospho-ERK1/2 (Cell signalling, Ipswich, MA, USA), Anti- phosphop-38 (Cell signalling, Ipswich, MA, USA) and total p38 MAPK (Cell signaling, Ipswich, MA, USA), JNK1/2 (Cell signaling, Ipswich, MA, USA) and ERK1/2. Anti-SAA (Santa Cruz Biotechnology, CA), Anti-Apo E (Millipore), Albumin (Santa Cruz Biotechnology, CA), and Anti- LBP (Santa Cruz, CA).

### Statistical Analysis

Statistical analyses were performed using the unpaired Student’s t-test. Error bars represent ± S.E.M. Kaplan-Meier curves were used to show survival over time. The data were analyzed using Graph Pad Prism software (version 5.0). *P < 0.05, **P < 0.01 and ***P < 0.001.

## Electronic supplementary material

Supplementary Information
