# Supplementary material for: Hepatocyte SHP-1 is a Critical Modulator of Inflammation During Endotoxemia
Source: Sci Rep. 2017 May 22;7:2218. doi: 10.1038/s41598-017-02512-7 (PMC5440389; doi:10.1038/s41598-017-02512-7)

Supplementary information

Hepatocyte SHP-1is a Critical Modulator of Inflammation
During Endotoxemia

Anupam Adhikari[1, 2], Caroline Martel[1, 2], André Marette[3] and Martin Olivier[1, 2]*

[1]Department of Medicine, Microbiology and Immunology, Faculty of Medicine, McGill University, Montréal, Québec, Canada; [2]The Research Institute of the McGill University Health Centre and Infectious Diseases and Immunity in Global Health Program, Montréal, Québec, Canada; [3]Heart and Lung Institute (Laval Hospital), Université Laval, Québec, QC, Canada.

*Corresponding author:
Martin Olivier, Ph.D.
1001 Boul. Décarie
The Research Institute of the McGill University Health Centre
Site Glen, Pavillon E/Block E
Montréal, Qc, H4A 3J1, Canada
E-mail: martin.olivier@mcgill.ca

Supplementary Figure 1.

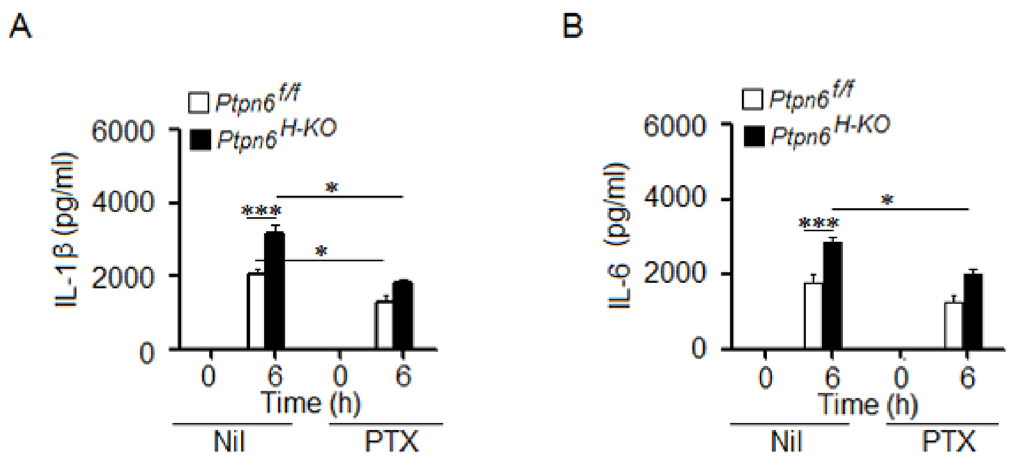

Supplementary Figure 1. TNF-α inhibitor pentoxifylline treatment reduced pro-inflammatory cytokines in *Ptpn6*$^{H\text{-}KO}$ challenged with LPS. Serum levels of (A) IL-1β and (B) IL-6 in *Ptpn6*$^{H\text{-}KO}$ and *Ptpn6*$^{f/f}$ mice treated with pentoxifylline, during LPS challenge (30 mg/kg body weight). Data are presented as mean ± SEM, $n = 6$. * $P \leq 0.05$, and ***$P \leq 0.001$.

Supplementary Figure 2

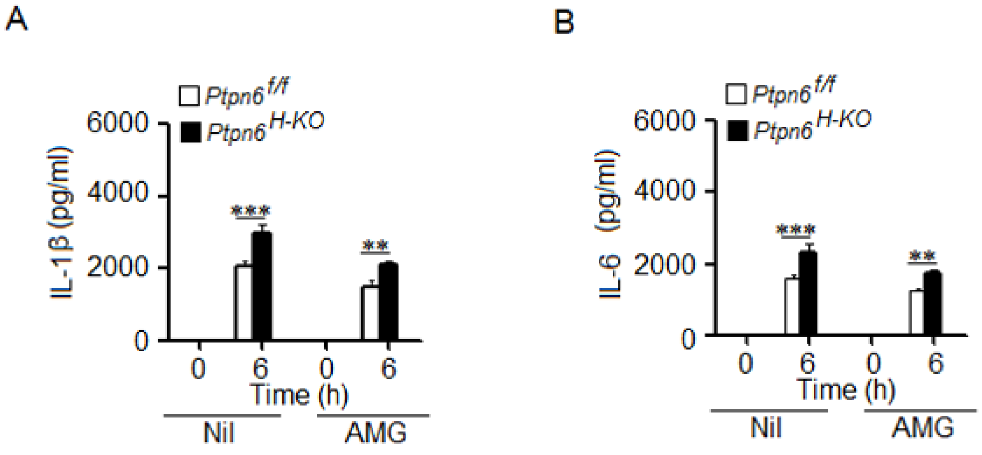

Supplementary Figure 2. Effect of iNOS/NO inhibitor aminoguanidine treatment on pro-inflammatory cytokines in *Ptpn6*$^{H\text{-}KO}$ challenged with LPS. Serum levels of (A) IL-1β and (B) IL-6 in *Ptpn6*$^{H\text{-}KO}$ and *Ptpn6*$^{f/f}$ mice treated with aminoguanidine, during LPS challenge (30 mg/kg body weight). Data are presented as mean ± SEM, *n* = 6 ***P ≤ 0.001.

Supplementary Figure 3

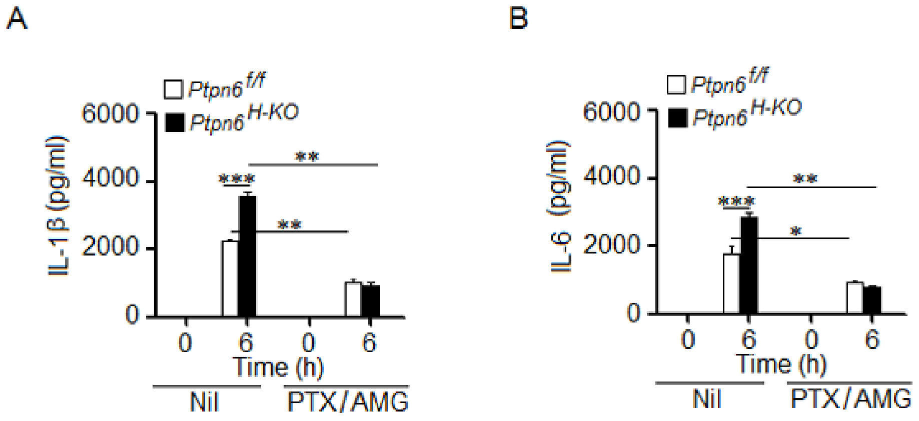

Supplementary Figure 3. Combination of TNF-α and iNOS/NO inhibitors treatment reduced pro-inflammatory cytokines in *Ptpn6*$^{H\text{-}KO}$ challenged with LPS. Serum levels of (A) IL-1β and (B) IL-6 in *Ptpn6*$^{H\text{-}KO}$ and *Ptpn6*$^{f/f}$ mice treated with aminoguanidine plus pentoxyfyline during LPS challenge (30 mg/kg body weight). Data are presented as mean ± SEM, $n = 6$. * $P \leq 0.05$, **$P \leq 0.01$ and ***$P \leq 0.001$.

Supplementary Figure 4.

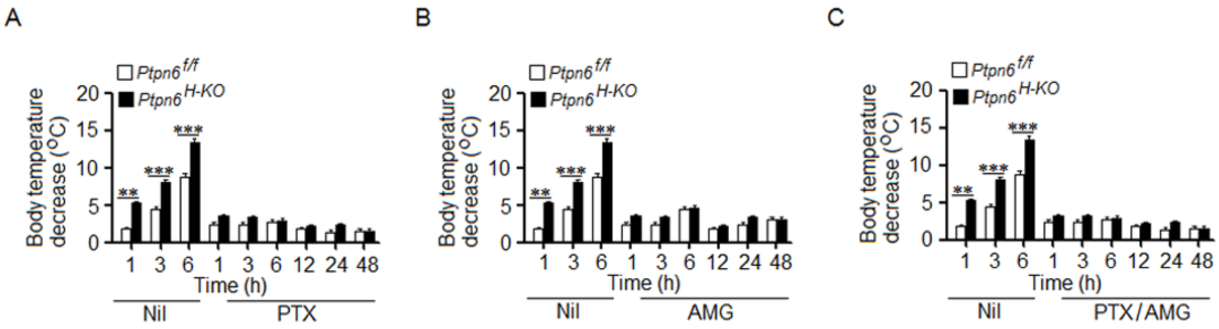

Supplementary Figure 4. Effect of TNF-α or NO inhibitors on body temperature of *Ptpn6*$^{H\text{-}KO}$ and the *Ptpn6*$^{f/f}$ mice Body temperature decrease in *Ptpn6*$^{H\text{-}KO}$ and *Ptpn6*$^{f/f}$ mice treated with (A) pentoxyfyline (B) amino guanidine and (C) their combination during LPS challenge (30 mg/kg body weight). No significant differences in body temperature reduction between *Ptpn6*$^{H\text{-}KO}$ and *Ptpn6*$^{f/f}$ mice at the indicated time points. Data are presented as mean ± SEM, $n = 6$. **$P \leq 0.01$ and ***$P \leq 0.001$.

Supplementary Information File 1

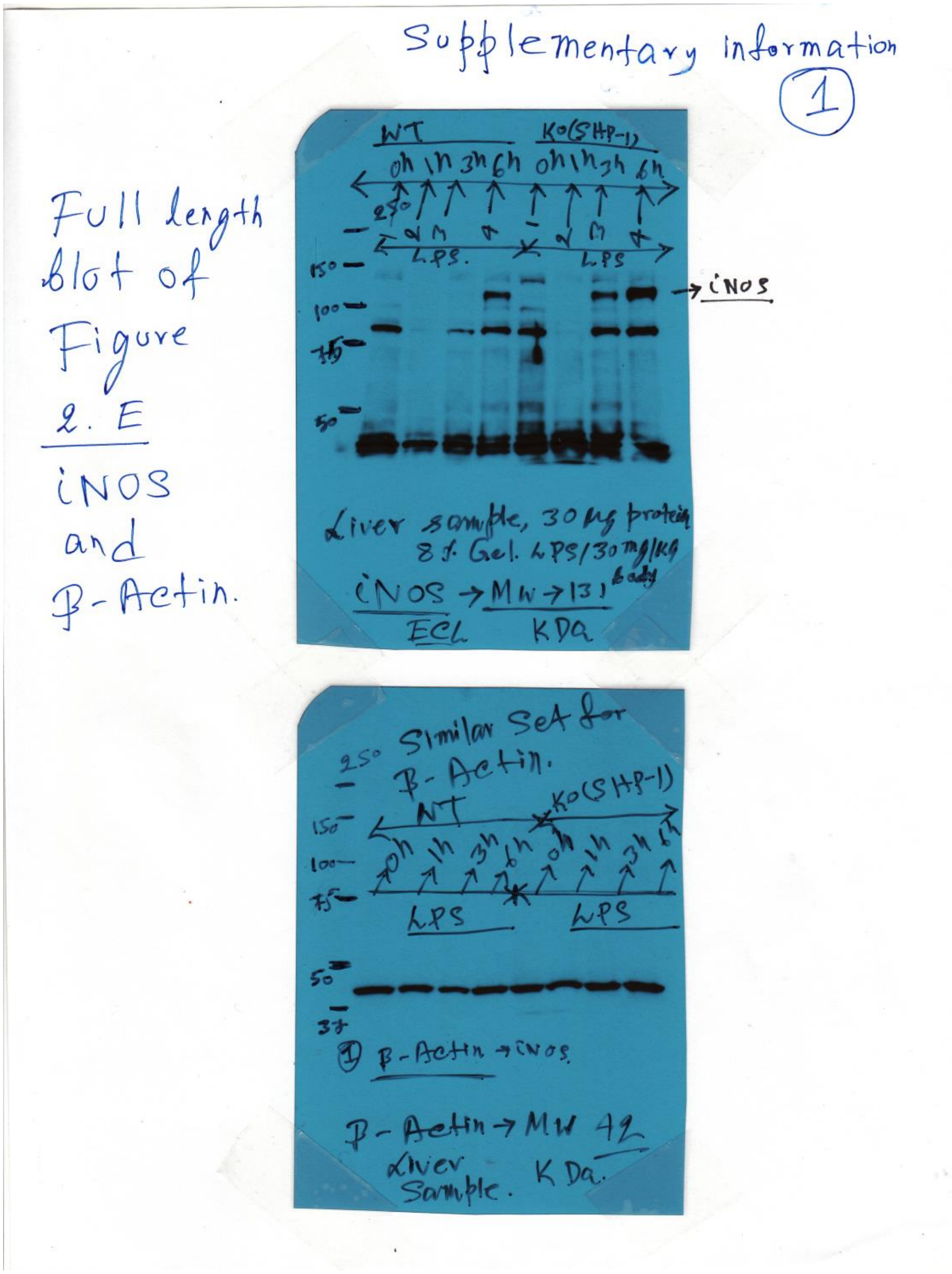

Supplementary Information File 2

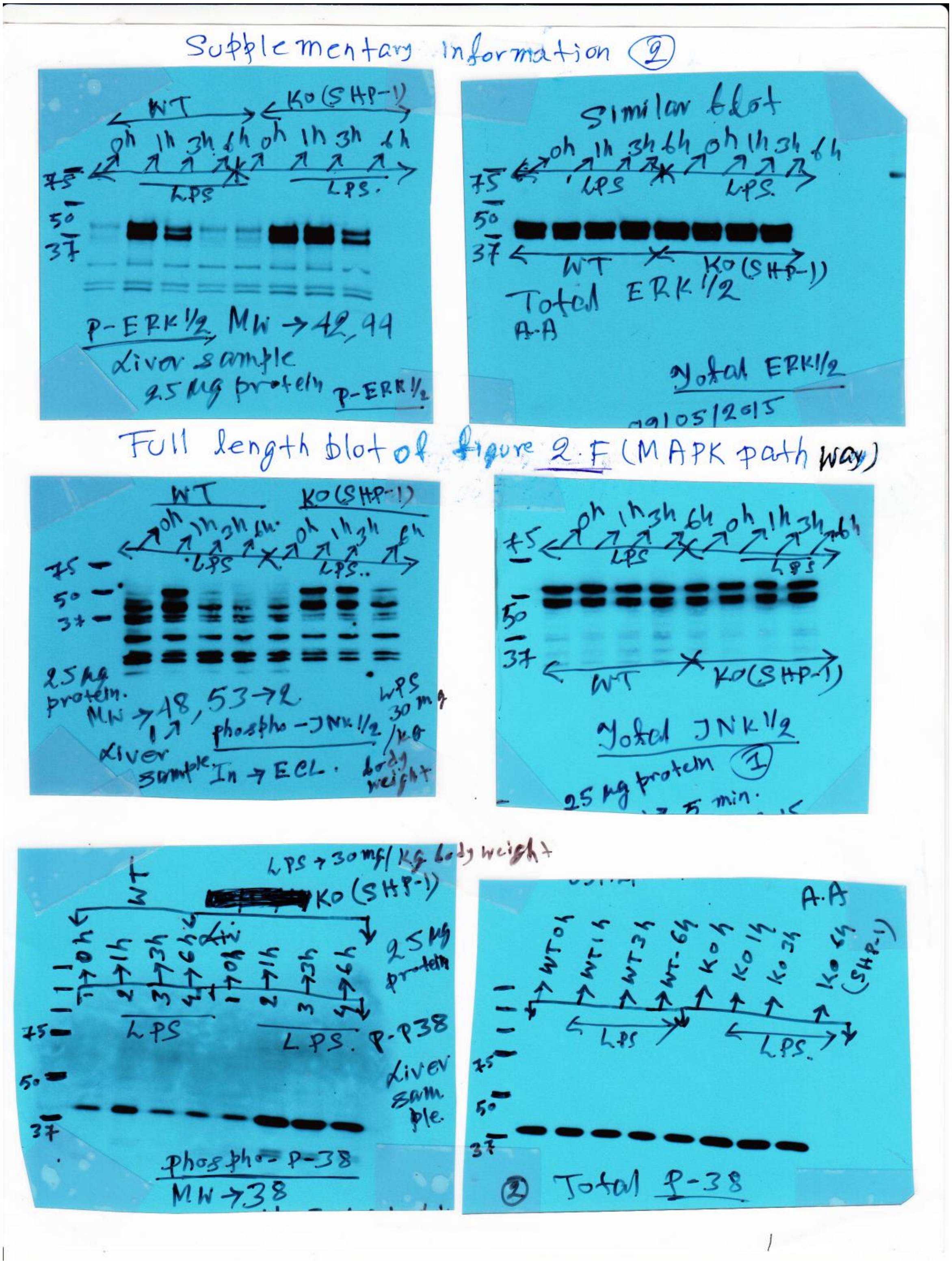

Supplementary Information File 3

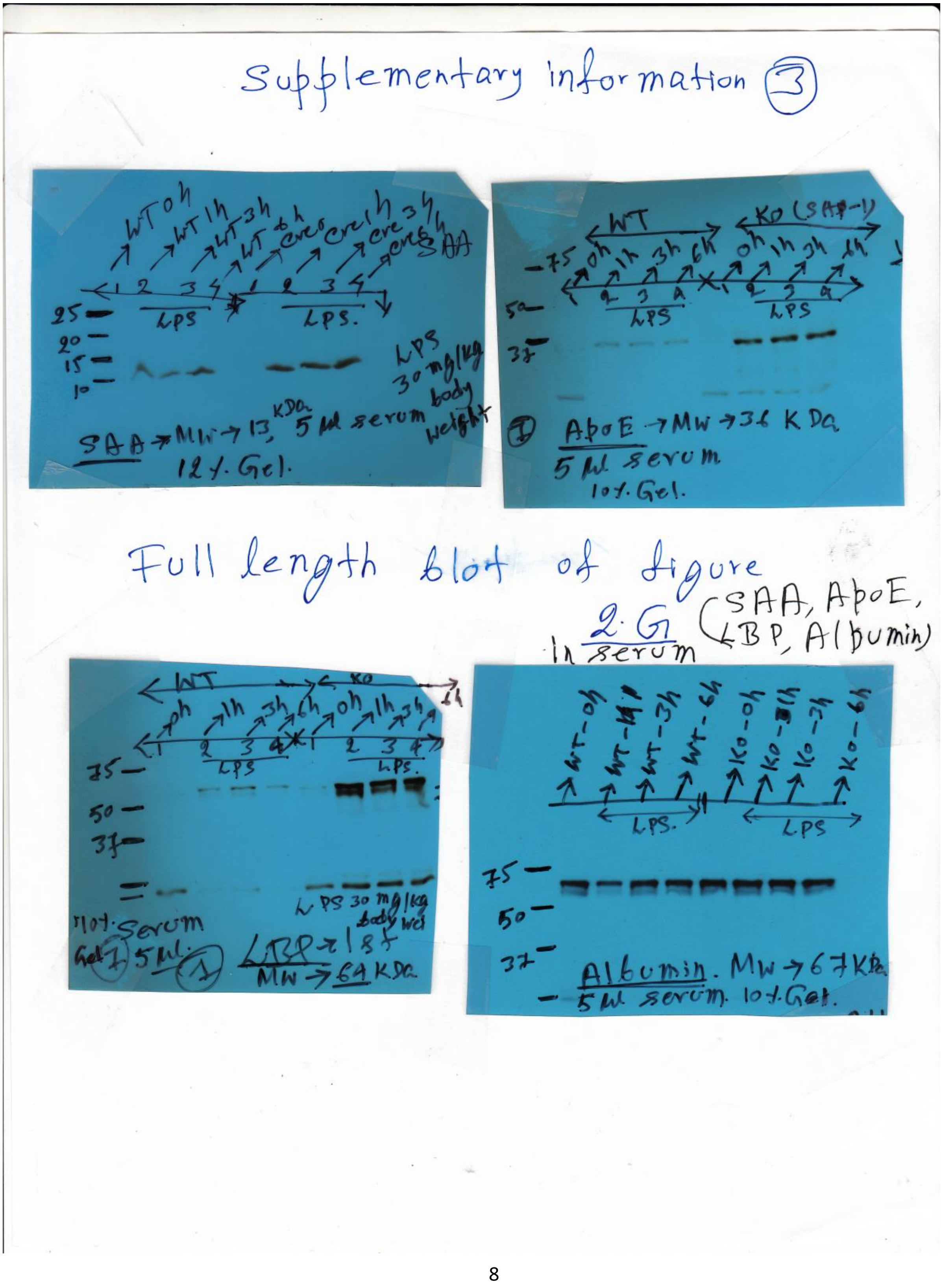

Supplementary Information File 4

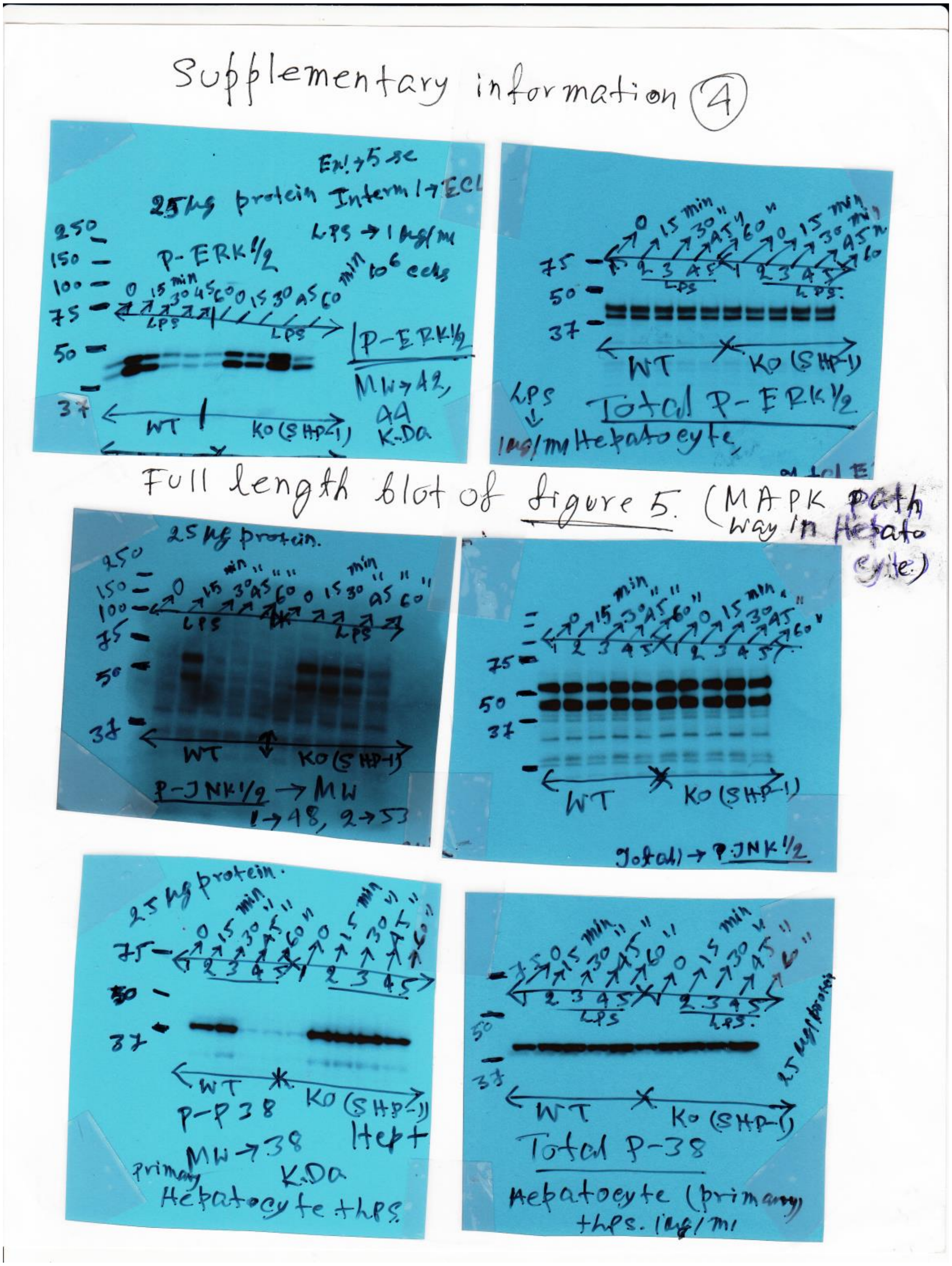

Supplementary Information File 5

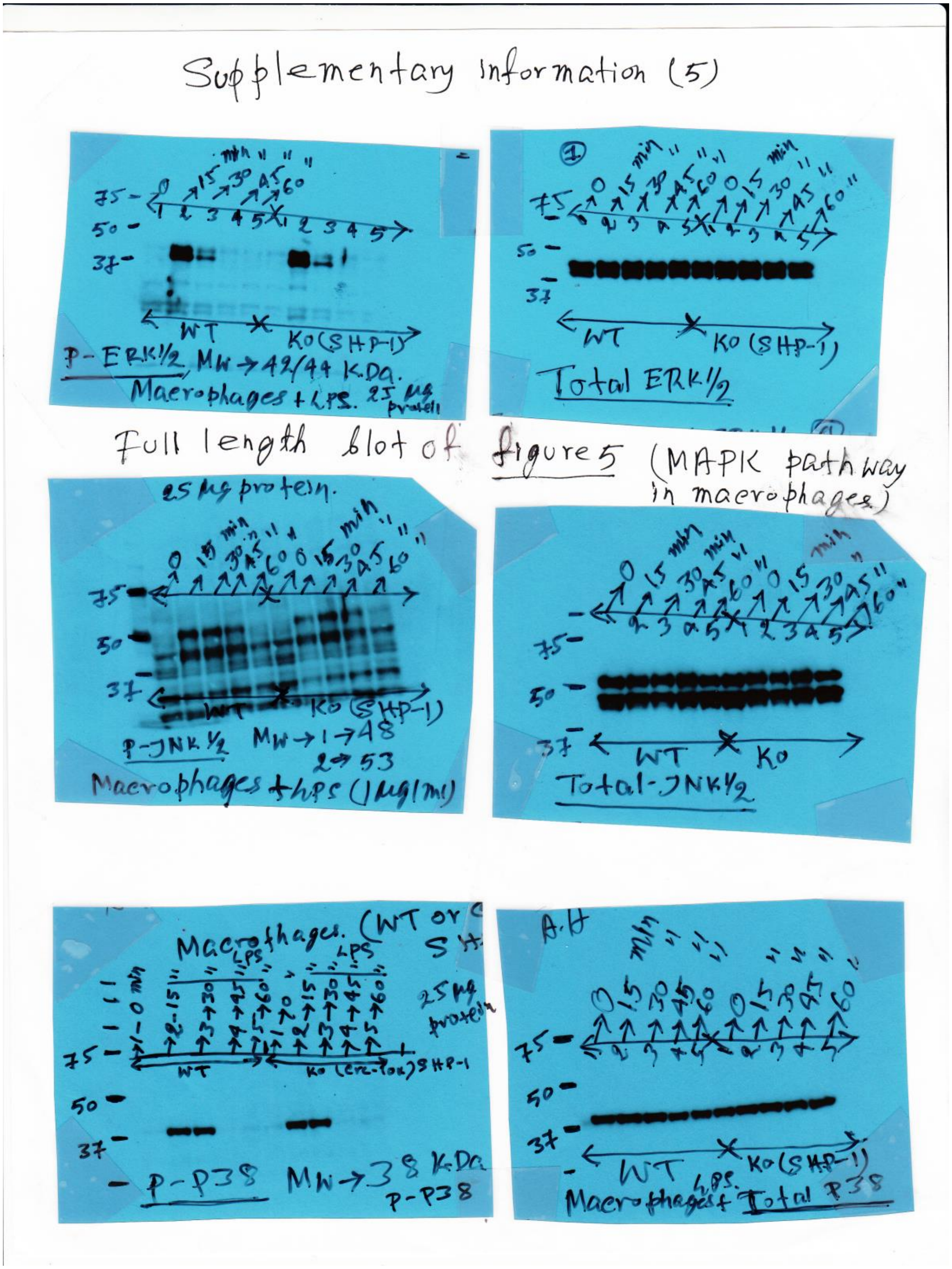

Supplement: Supplementary file 1 — Supplementary Information [file 41598_2017_2512_MOESM1_ESM.pdf]
